# Microstructural Evolution of Hybrid Perovskites Promoted by Chlorine and its Impact on the Performance of Solar Cell

**DOI:** 10.1038/s41598-019-41328-5

**Published:** 2019-03-18

**Authors:** Byungho Lee, Taehyun Hwang, Sangheon Lee, Byungha Shin, Byungwoo Park

**Affiliations:** 1 Department of Materials Science and Engineering, Research Institute of Advanced Materials, Seoul National University, Seoul, 08826 Korea; 2 Department of Materials Science and Engineering, Korea Advanced Institute of Science and Technology, Daejeon, 34141 Korea

## Abstract

The role of Cl in halide hybrid perovskites CH_3_NH_3_PbI_3_(Cl) (MAPbI_3_(Cl)) on the augmentation of grain size is still unclear although many reports have referred to these phenomena. Herein, we synthesized MAPbI_3_(Cl) perovskite films by using excess MACl-containing precursors, which exhibited approximately an order of magnitude larger grain size with higher <110>-preferred orientation compared with that from stoichiometric precursors. Comprehensive mechanisms for the large grain evolution by Cl incorporation were elucidated in detail by correlating the changes in grain orientation, distribution of grain size, and the remaining Cl in the perovskite during thermal annealing. In the presence of Cl, <110>- and <001>-oriented grains grew faster than other grains at the initial stage of annealing. Further annealing led to the dissipation of Cl, resulting in the shrinkage of <001> grains while <110> grains continuously grew, as analyzed by x-ray rocking curve and diffraction. As a result of reduced grain boundaries and enhanced <110> texture, the trap density of perovskite solar cells diminished by ~10% by incorporating MACl in the precursor, resulting in a fill factor more than 80%.

## Introduction

An organic-inorganic hybrid perovskite solar cell has emerged as a most promising photovoltaic among next-generation solar cells owing to their proper optical/electronic properties resulting in the photovoltaic efficiency over 23%, with low loss of open-circuit voltage and low-temperature processability reducing the production cost^1–6^. Particularly, the role of Cl in the perovskite solar cells has drawn tremendous attention in light of its intriguing and effective impacts on improving the device performance. The most common method of incorporating Cl into CH_3_NH_3_PbI_3_ (MAPbI_3_) is adding Cl-containing precursors such as PbCl_2_ or MACl for the spin-coating process. However, the vast majority of Cl in the as-spun films is dissipated from the films during thermal annealing through sublimation and/or decomposition^7–9^: Cl which manages to remain in the final films is mainly observed at grain boundaries, and in the vicinity of the perovskite/TiO_2_ interface in the case of *n*-*i*-*p* solar cells with TiO_2_^10–13^. The maximum amount of Cl that can be incorporated into MAPbI_3_ via conventional spin-coating process has been reported to be limited to <4 at % (chlorine vs. iodine) due to the substantial differences in ionic radii^14^. Despite the small amount present in the perovskite films, incorporation of Cl has been shown to significantly improve the performance of perovskite solar cells^15^.

Two major known benefits of the Cl incorporation are in improving the optoelectronic properties and microstructures of MAPbI_3_. Cl has been demonstrated to passivate defects in surfaces, grain boundaries, and interfaces, therefore, suppressing parasitic nonradiative recombination^16–20^. For example, theoretical studies have shown that Cl present at the perovskite/TiO_2_ interface reduces deep-level defects by substituting Pb-I antisites with Pb-Cl antisites which have higher formation energy with shallower level^19^. In terms of the microstructural modification, Cl significantly influences the formation process of perovskite films. Changes in the chemistry of Pb-halide ionic complexes and colloids with PbCl_2_ or MACl precursor has been shown to enable high coverage of perovskite films^21,22^. Additionally, intermediate phases containing Cl, which eventually transform into MAPbI_3_ upon thermal annealing, slow down the reaction kinetics contributing to the enhanced coverage^7,23,24^. After thermal annealing, large lateral grain size exceeding ~1 μm is frequently accompanied with high <110>-preferred orientation in MAPbI_3_(Cl) films regardless of the Cl source^25–29^. Although it has been well known that Cl develops large grains in the perovskite films, a mechanistic study revealing the detailed roles of Cl in the film growth kinetics and the correlation between enhanced grain size, crystallinity and concentration of Cl in the film still lacks.

Herein, we demonstrate the mechanisms of large grain growth in MAPbI_3_(Cl) films synthesized with excess-MACl containing precursors. Apparent correlation between the changes in preferred orientation, distribution of grain size, and the amount of remaining Cl in the films is observed elucidating the effect of Cl on the formation processes. At the initial stage of annealing with Cl in the films, <110>-  and <001>-oriented grains grow faster than other grains. Extended annealing time causes dissipation of Cl, and <001> grains become unstable while <110> grains continuously grow. As a result, grains much larger than—by an order of magnitude—those from stoichiometric precursors are obtained with an average grain size exceeding 2 μm and highly <110>-preferred orientation. Due to the improved texture and diminished deep trap density, the fill factor of solar cells reached over 80%. We expect that these findings will provide further guidance on how to control the morphology and grain orientation of perovskite films with Cl.

## Results and Discussion

**Figure 1:**
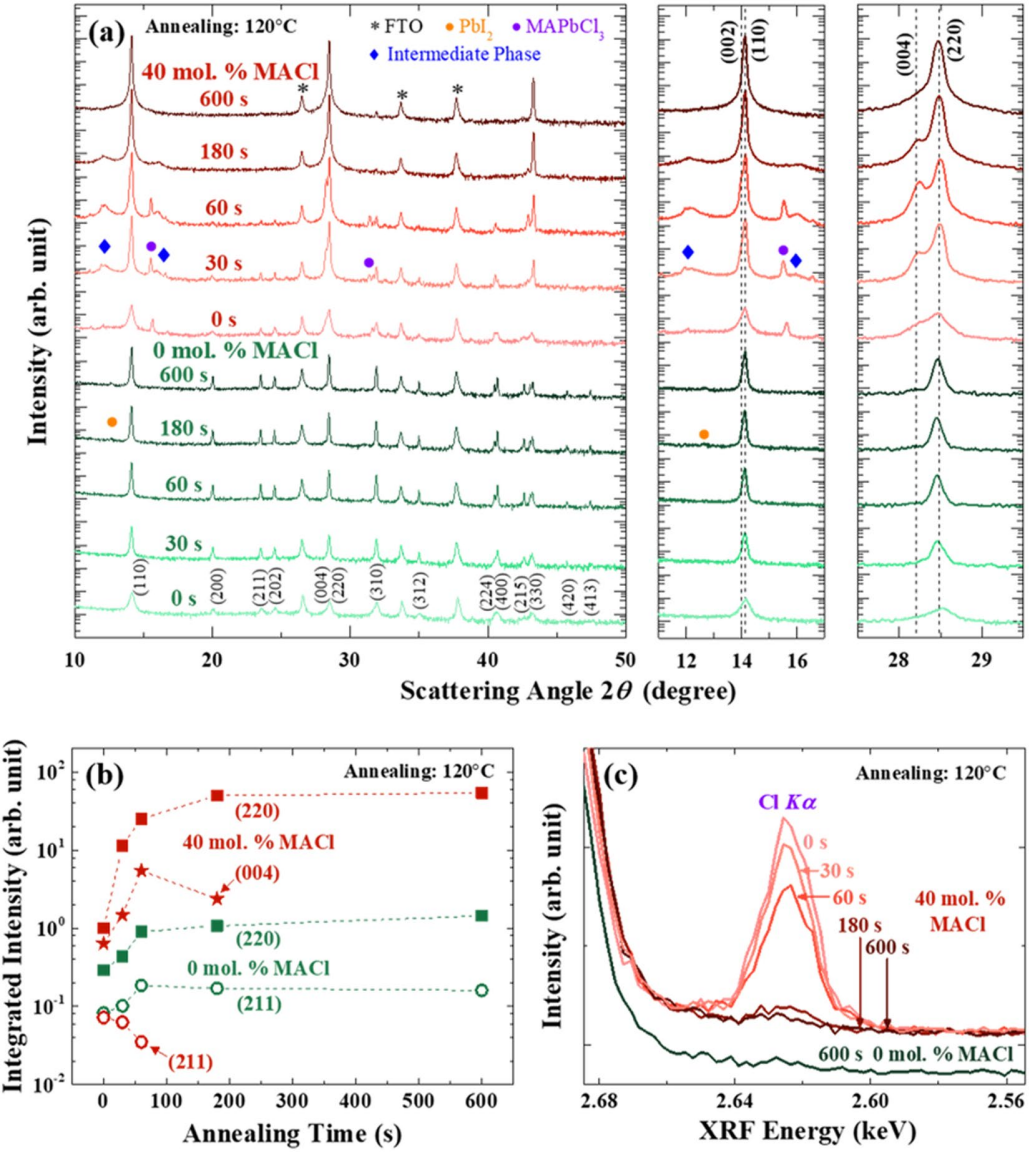
Grain orientations and remaining Cl with different annealing times at 120 °C. (**a**) X-ray diffraction, (**b**) relative integrated intensities of (220), (004), and (211) peaks, and (**c**) XRF spectra of Cl from the perovskite films. Precursor compositions are PbI_2_:MAI:MACl = 1:1:0.4 (40 mol. % MACl) or PbI_2_:MAI:MACl = 1:1:0 (0 mol. % MACl).

The perovskite films are spin-coated with the antisolvent dripping method to ensure uniform coverage^2,30^. In the case of perovskite with stoichiometric precursor, intensities of all of the perovskite peaks are enhanced with annealing, and minor PbI_2_ peak appears at 12.7° after annealing for 180 s indicating partial thermal decomposition of the perovskite, as shown in the log-scale diffraction patterns of Fig. 1(a). With the 40-mol.-% MACl additive, peaks from MAPbCl_3_ at 15.5° and 31.4° and from intermediate phase related to the excessive Cl at around 12.0° and 16.0° are observed^7,24,31,32^. The intensities of these peaks increase with annealing for 60 s, weaken at 180 s, and eventually disappear after 600 s. As far as peaks from MAPbI_3_ are concerned, the as-deposited perovskite with MACl-containing precursor exhibits almost identical patterns with the perovskite from the stoichiometric precursor, except slightly higher <110>-preferred orientation with intermediate phases (Fig. S1). After annealing for 600 s, however, the degree of preferred orientation is significantly altered compared to the non-annealed films. As shown in Fig. 1(b), integrated intensity of (220) peak of the 600-s annealed perovskite using MACl-containing precursor enhances by 50 times compared to the as-deposited film, while intensity enhancement by annealing is much weaker for the perovskite with stoichiometric precursor (only ~5 times increase). Especially, the intensity mainly rises during the first 30 s of annealing. In addition, the integrated intensity of (211) peak based on the stoichiometric precursor increases during annealing with similar tendency to (220) peak, while it gradually reduces and eventually disappears in the perovskite with the MACl precursor. This intensity weakening by annealing occurs in other MAPbI_3_ peaks in a similar manner except <110> and <001> peaks. The intensity of (004) peak, which is hardly observed from the stoichiometric-precursor-based perovskite, increases during the first 60 s of annealing in the perovskite with MACl-containing precursor. However, further annealing beyond 180 s reduces the (004) peak intensity while (110) peak intensity intensifies.

It has been reported that excess MACl in the as-spun state of a perovskite film dissipates during annealing by sublimation or via decomposition into CH_3_NH_2_ and HCl^7,8^. While Pb *Lβ*_1_ peak intensity remains nearly constant with annealing (Fig. S2), the intensity of Cl *Kα* gradually decreases and becomes below the noise level after 600 s, as confirmed by the XRF spectra vs. annealing time in Fig. 1(c). It is noted that the large reduction of Cl after 180 s is accompanied by the weakening of XRD intensities of the intermediate phase and MAPbCl_3_ phase (see Fig. 1(a)), which suggests that the main source of Cl signal is the intermediate phase or MAPbCl_3_. Also, there are correlations between the integrated intensity of MAPbI_3_ in XRD ((220) and (004) peaks) and the remaining amount of Cl. With the reduction of Cl, growth of (220) peak intensity is slowed down, and the (004) peak becomes negligible after 600 s.

**Figure 2:**
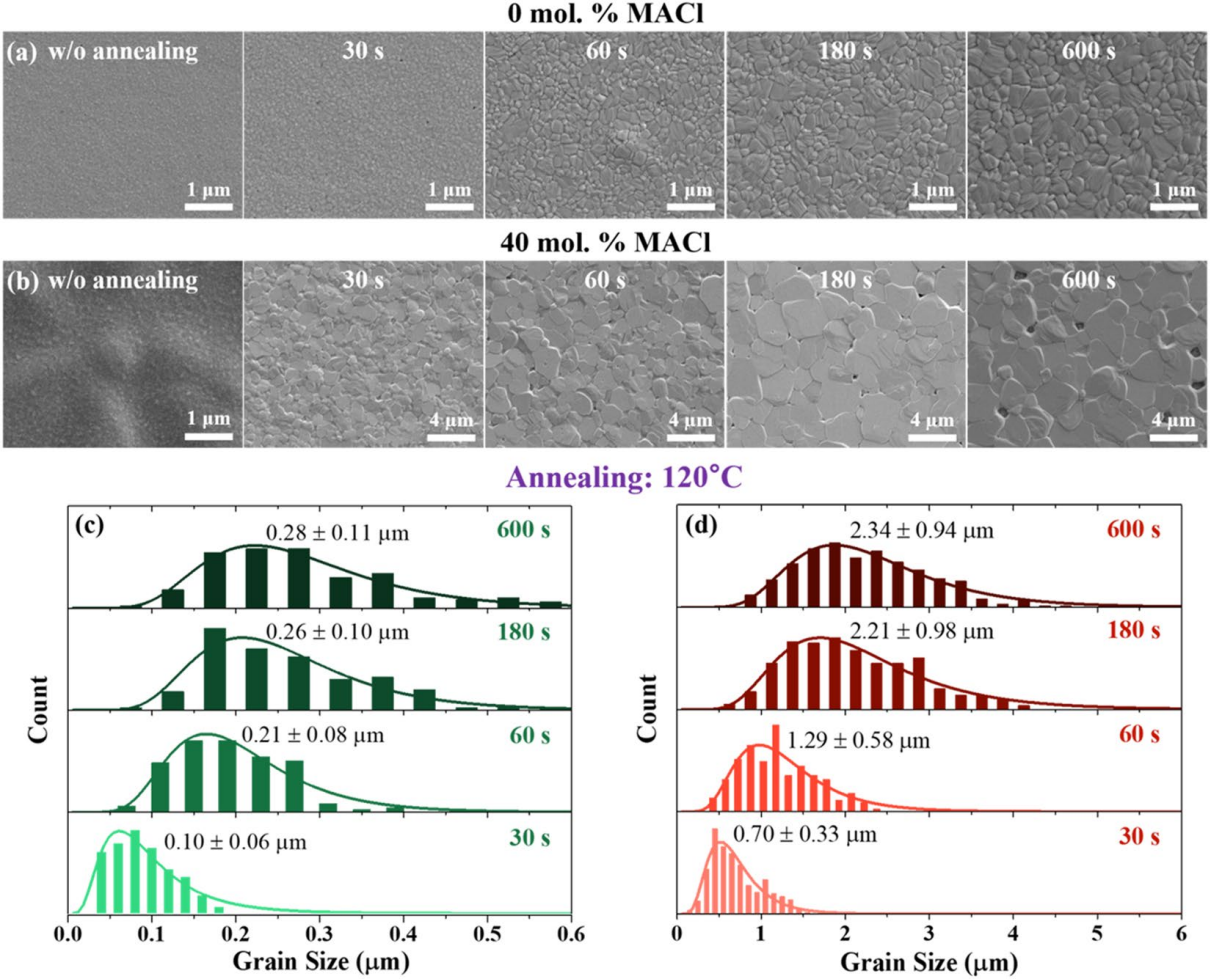
Grain size analyses. Evolution of grains in the perovskite films (by SEM) for (**a**) stoichiometric precursor (0 mol. % MACl) and (**b**) MACl-containing precursor (40 mol. % MACl) with different annealing times. (**c**,**d**) Distributions of grain sizes for the perovskites with fitting lines. Grain sizes without annealing are plotted in Fig. S3.

The effects of annealing on the grain growth are significantly different depending on the precursor composition, as shown in SEM (Fig. 2(a,b)). For the as-deposited films, both perovskites exhibit similar grain sizes of ~50 nm (Fig. S3). In the case of perovskite from the stoichiometric precursor, an average grain size of ~300 nm is achieved after 180-s annealing (Figs 2(c) and S3). The lateral grain size less than film thickness (~450 nm) may result from the Zenner pinning induced by PbI_2_ located at the grain boundary, formed after 180 s of annealing from the thermal decomposition of perovskite. However, the PbI_2_ cannot be observed in the SEM images of perovskite until annealing for 600 s, presumably due to its small size. When annealing was prolonged up to 30 min, preferential formation of PbI_2_ particles at the grain boundaries is clearly observed: PbI_2_ particles appear as bright dots in the SEM image owing to the insulating nature (Fig. S5).

The grain growth rate of perovskite based on the MACl-containing precursor is significantly enhanced compared to the stoichiometric case (Fig. 2(d)). During the first 30 s of annealing, average grain size increases from 50 nm to 700 nm. When we compare the average grain size with the integrated intensity in Fig. 1(b), increase in the grain size for 60 s accompanies the intensified (220) and (004) peaks while other randomly oriented peaks such as (211) diminish. Therefore, the augmentation of the average grain size can be mainly attributed to the growth of <110> and <001> grains. The faster growth kinetics of <110> and <001> grains in the presence of Cl may be explained by a recent computational study which showed that Cl at the MAPbI_3_/TiO_2_ interface reinforces the binding of (110) and (001) planes of MAPbI_3_ with TiO_2_^33^. During annealing from 60 to 180 s, Cl is largely dissipated as evident from XRF in Fig. 1(c) with further increase in (220) and reduction of (004) peak in XRD. However, grain growth rate is still faster than the stoichiometric-precursor perovskite, and eventually slows down after 180 s annealing (Fig. S3). After annealing for 600 s, the final average grain size reaches ~2 μm which is much improved compared to the perovskite with stoichiometric precursor.

**Figure 3:**
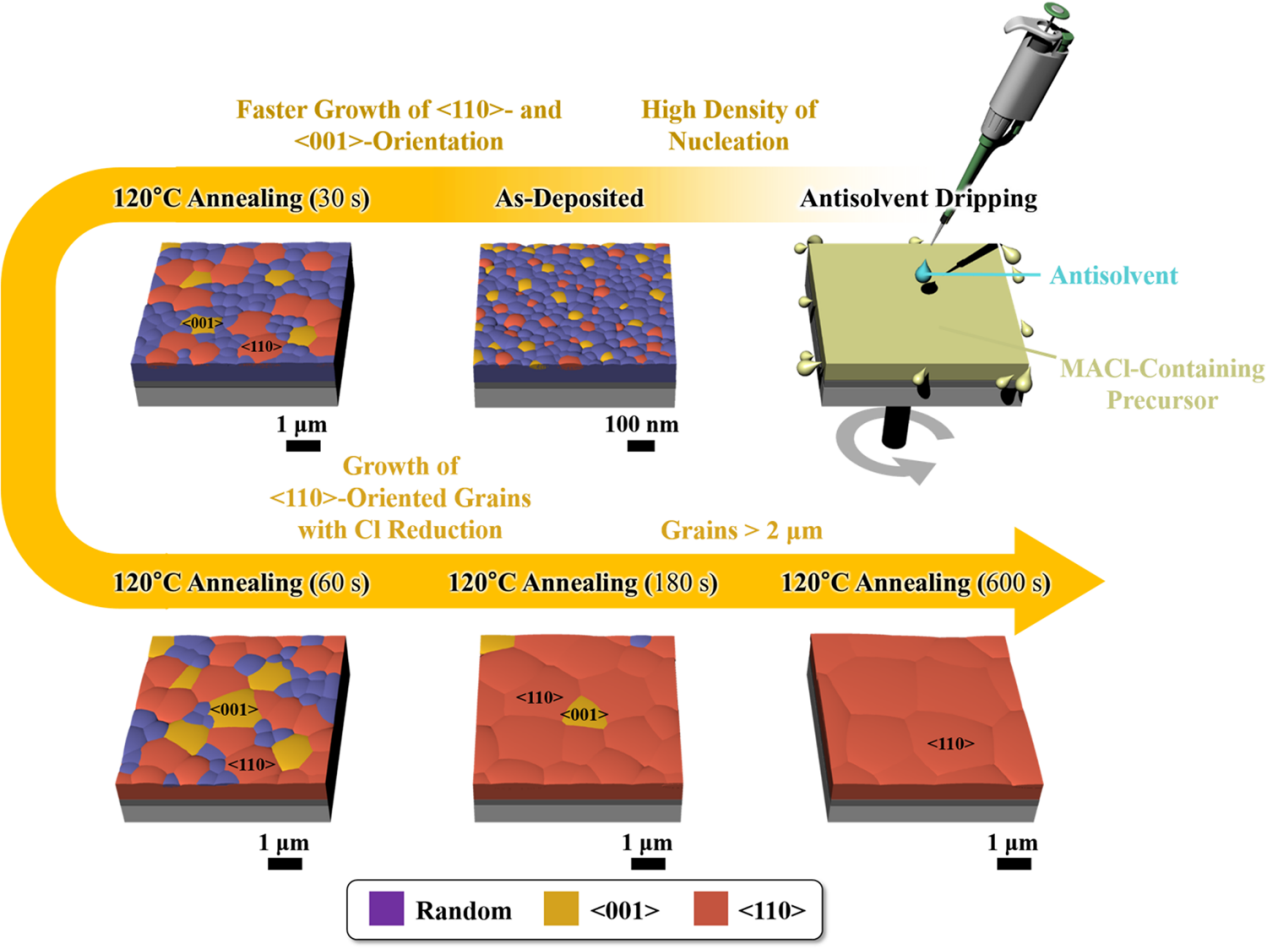
Schematic diagram for the microstructural evolution of perovskite films with MACl-containing precursor.

Combing XRD, XRF, and SEM data, microstructural evolution of the perovskite from MACl-containing precursor is schematically illustrated in Fig. 3. A high density of nuclei is formed by the antisolvent dripping. During the first 30 s of annealing, the growth rates of <110>- and <001>-oriented grains are faster than other orientations. During the continued annealing for 60 s, <110> and <001> grains continue to grow until they are in contact with each other. Further annealing leads to the reduction of Cl content in the film, and <110> grains further grow over the 2-μm grain size at the expense of neighboring random and <001> grains.

Evolution of structural and chemical properties of the perovskite from the 20-mol.-% MACl-containing precursor is presented in Fig. S4. The evolution of the grain-size distribution and orientation is qualitatively similar to the case of 40-mol.-% MACl, with a relatively faster evolution. MAPbCl_3_ and intermediate phase are observed up to 60-s annealing and disappear after 180 s from XRD. The intensities of (220) and (004) peaks of MAPbI_3_ significantly increase during the first 30 s of annealing accompanied by the rapid grain growth as can be seen from the SEM images. On the other hand, the intensity of (004) peak decreases after 60 s, and finally disappears after 180 s. XRF spectra exhibits reduced intensity of Cl *Kα* with the prolonged annealing and negligible signal after 180-s annealing, which is consistent with the dissipation of Cl-related phase from XRD. In addition, disappearance of (004) peak with complete loss of Cl in the film is analogous to the case of 40-mol.-% MACl. These results reveal that rapid texturing of <110>- and <001>-oriented grains in the presence of Cl followed by the growth of <110>-oriented grains, at the expense of <001>-oriented grains once Cl is dissipated.

**Figure 4:**
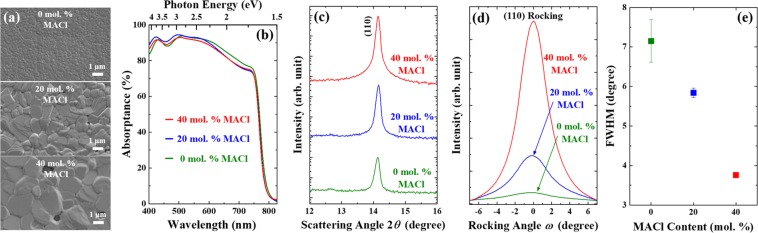
Effects of MACl concentration on the morphology, light absorption, and texture of the perovskite films. (**a**) Grain sizes by SEM and (**b**) absorptance spectra. X-ray diffraction with (**c**) *θ*–2*θ* scan, (**d**) *ω* scan for the (110)-plane rocking, and (**e**) FWHM of rocking curves from (**d**). All the films are annealed at 120 °C for 600 s.

The larger amount of MACl in the precursor has induced larger grain size, as shown in Fig. 4(a) after 600-s annealing. While light absorption characteristics do not change much by the MACl content (Fig. 4(b)), the <110>-peak intensity is the highest with the addition of 40% MACl to the precursor solutions (Fig. 4(c)). From *ω* scans of perovskite (110) plane, both the intensity and full-width at half maximum (FWHM) improve with the increasing MACl content (Fig. 4(d,e)), again confirming the enhanced <110> texture of the perovskite films. In addition, steady-state and time-resolved PL spectra of the perovskite from differing amount of Cl in the precursor solutions are compared in Fig. S6. With the addition of MACl, the emission peaks of perovskites shift to shorter wavelength suggesting the reduced trap states (band tails). Time-resolved PL spectra are fitted to a bi-exponential decay curve, and the extracted fast (*τ*_1_) and slow (*τ*_2_) decay components are listed in Fig. S6. The lifetimes improve with the increasing MACl contents in the precursor supporting the suppression of defect-mediated nonradiative recombination. The enhanced PL characteristics can be attributed to the reduced grain boundaries and enhanced <110> texture of perovskite films^16^. While 60-mol.-% MACl makes grain sizes over 3 μm, the coverage of a perovskite film is only ~90%, and therefore these results are not included here.

**Figure 5:**
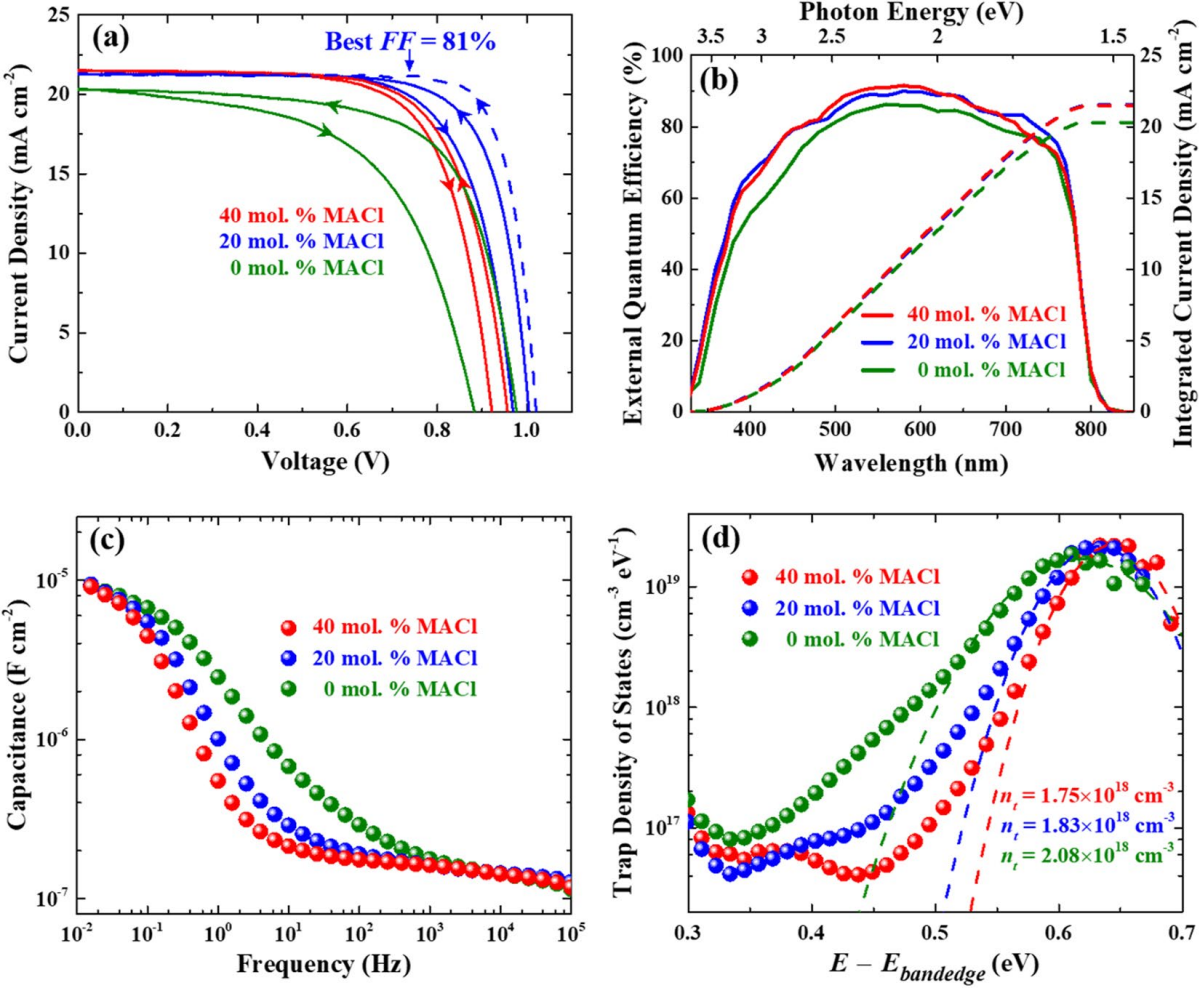
Solar-cell performance and trap-density characterization. (**a**) *J−V* curves at both reverse and forward voltage scans. The blue dashed line represents the highest efficient cell. (**b**) Incident photon-to-current conversion efficiency (IPCE). (**c**) Frequency dependent capacitances (*C*(*ω*) ≡ *Real* [{*iωZ*(*ω*)}^−1^]) obtained from the impedance analysis. (**d**) Trap density of states as a function of trap level with respect to the valance-band maximum, from Eqs (1) and (2). The dashed lines denote Gaussian fitting for the calculation of traps per volume (*n*_*t*_). All the perovskite films are annealed at 120 °C for 600 s.

Figure 5(a) exhibits *J-V* characteristics of the perovskite solar cells (with the detailed photovoltaic parameters in Table S1). Addition of 20-mol.-% MACl largely improves the short-circuit current (*J*_*sc*_) and fill factor (*FF*), and the best photovoltaic efficiency of *η* = 17.7% with a *FF* of 81% is achieved. In the case of 40-mol.-% MACl, *V*_*oc*_ and *FF* decrease while *J*_*sc*_ is similar to those of the 20-mol.-% MACl. Series and shunt resistances are extracted by fitting the light *J-V* curves to the ideal one-diode model (Fig. S7)^34,35^. Series resistance from the 20-mol.-% MACl cell is much better (0.5 Ω·cm^2^) than that of the stoichiometric-precursor-based cell (1.7 Ω·cm^2^). A cross-sectional SEM image exhibits that horizontal grain boundaries are hardly observed from the 20-mol.-% MACl solar cell, and the shunt resistance is the highest which we attribute to the large grains without pin-holes (Fig. S8)^36,37^. More MACl induces incomplete perovskite coverage, thereby reducing the shunt resistance. In addition to the enhanced photovoltaic efficiency, *J-V* hysteresis is improved by incorporating MACl, presumably due to the suppressed defects and ion migration in the synthesized perovskites with the electron/hole transport layers (Table S1)^38,39^. Furthermore, stabilized power outputs for the cells based on MACl-containing precursors exhibit similar values to their average efficiency of reverse and forward scans in the *J-V* curve with relatively stable output during the measurement, while the solar cell from the stoichiometric precursor shows poor stability (Fig. S9). EQE of the perovskite solar cells based on various concentrations of MACl clearly show different spectral responses (Fig. 5(b)) and the integrated current of each spectra matches well to the observed *J*_*sc*_. EQE of solar cells with 20-mol.-% MACl is higher compared to that of the stoichiometric perovskite cell for the entire wavelength range. It is noted that annealing time shorter than 10 min yield poorer efficiency probably due to the smaller grain size and presence of the intermediate phase which may prevent carrier transport (Fig. S10). With the larger amount of MACl, the solar cells become more stable in air due to the reduced grain boundary, which is suggested to be the main degradation routes to react with H_2_O and O_2_ (Fig. S11)^40,41^. The efficiencies of solar cells based on the MACl-containing precursors maintain over 90% of their initial values after 20 days exhibiting stable device performance.

To quantify the trap density in the perovskite solar cells, impedance spectroscopy measurements are carried out. By analyzing the frequency dependent capacitances [*C*(*ω*) ≡ *Real*[{*iωZ*(*ω*)}^−1^]], trap levels with respect to the valence-band maximum and trap density can be extracted^42^. As shown in Fig. 5(c), the capacitance plot exhibits similar high frequency plateau irrespective of the MACl contents and different rising level in the frequency region dependent on the amount of MACl. The rate of capture and emission of charge carriers by trap states depends on the level of the trap states with respect to the band edge, and charging/discharging of these trap states contributes to measured capacitance. Only shallow traps, which have been shown to reside in the bulk of perovskite^43,44^, respond to high-frequency modulation, and deep traps at interfaces or grain boundaries can be responsible at relatively low frequency resulting in the capacitance rise. Figure 5(d) compares distributions of the trap density of state extracted using the derivative of the capacitance with respect to the frequency:

1 $${N}_{t}=-\,\frac{{{V}}_{{bi}}}{{qW}}\frac{{dC}}{d{\omega }}\frac{\omega }{{k}_{B}T}$$

where *V*_*bi*_ is the built-in potential, *q* is the elementary charge, *W* is the depletion width, *ω* is the angular frequency, *k*_*B*_ is the Boltzmann constant, and *T* is temperature^42,45^. The built-in potentials are obtained from the Mott-Schottky plots (Fig. S12), and perovskites are assumed to be fully depleted and hence are considered as an intrinsic layer^46^. The applied angular frequency is related to a trap level with respect to the valence-band maximum (*E*_*ω*_):

2 $${E}_{\omega }={k}_{B}{T}\,\mathrm{ln}(\frac{{\omega }_{0}}{\omega })$$

where *ω*_0_ is an attempt-to-escape frequency, and assumed to be ~10^11^ s^−1^^46,47^. The integrated defect densities of MACl-containing cells (20 mol. %: 1.83 × 10^18^ cm^−3^, 40 mol. %: 1.75 × 10^18^ cm^−3^) reduce by ~10% compared to that of a stoichiometric-precursor-based perovskite cell (2.08 × 10^18^ cm^−3^). These defect reduction can be attributed to the improved grain boundaries and interfaces where deep-level defects like Pb-I antisites are segregated in the perovskite film^48,49^ and eventually results in the enhanced photovoltaic performances.

## Conclusions

In this work, it is shown that inclusion of Cl in the precursor solutions leads to large grain size. At the initial stage of annealing when a large amount of Cl remained, <110>- and <001>-oriented grains grew faster than others. With further annealing which reduced the Cl content, <110> grains were further coarsened at the expense of intermediate phase and other perovskite grains. Grain size over 2 μm, almost an order of magnitude larger than the case of stoichiometric precursor, was achieved. In addition, texturing of the perovskite films was enhanced with the increased MACl, as evident from the narrowing of rocking curves. Applications of the perovskites with the optimized concentration of MACl in the precursor solutions to the light absorber improved resistance of the devices resulting in a fill factor over 80%. Deep trap density was reduced from 2.08 × 10^18^ cm^−3^ (no Cl) to 1.83 × 10^18^ cm^−3^ (with Cl). Our study elucidates correlation between commonly reported <110>-preferred orientation and large grain sizes with Cl in the films, which will further help with better microstructural design of the perovskites.

## Experimental Procedures

### Device Fabrication

Unless stated otherwise, all chemicals were purchased from Sigma-Aldrich. Fluorine-doped tin oxide (FTO, TEC 8: Pilkington) glasses were ultrasonically cleaned in acetone, ethanol, and deionized water, followed by UV-ozone treatments for 15 min. For TiO_2_ blocking layer, 0.25 M titanium isopropoxide and 0.44 M acetic acid (DAEJUNG) in ethanol was spin-coated at 3000 rpm for 30 s, and annealed at 500 °C for 30 min. The substrates were dipped in 40 mM TiCl_4_ aqueous solutions at 70 °C for 30 min, followed by annealing at 500 °C for 30 min. The precursor solutions for the perovskites were prepared with 1.4 M of equimolar PbI_2_ (TCI) and CH_3_NH_3_I (Greatcell Solar) in *N*,*N*-dimethylformamide (DMF), and 0.2, 0.4, or 0.6 molar ratio of CH_3_NH_3_Cl relative to CH_3_NH_3_I was introduced as an additive. The solutions were deposited onto the substrates by spin coating at 5000 rpm for 20 s, and 300 μL of chlorobenzene was dripped at 5 s into the spinning process. Then, the films were annealed at 120 °C for 10 min. The hole transporting layers were deposited by spin-coating at 3000 rpm for 45 s using the precursor solutions prepared by mixing 72.3 mg/ml spiro-OMeTAD (Lumtec) in chlorobenzene with 28.8 µL of 4-*tert*-butylpyridine, a 17.5 µL of lithium *bis*(trifluoromethylsulfonyl)imide (Li-TFSI) solution (520 mg/ml in acetonitrile), and a 21.9 µL of Co(II)-TFSI (Lumtec) solution (300 mg/ml in acetonitrile). Finally, 150 nm-thick Au electrodes were deposited by thermal evaporation.

### Characterization

The crystal structure and texture of perovskite films were analyzed by x-ray diffraction (XRD) in *θ*−2*θ* scan mode (D8 Advance: Bruker) and rocking curve mode (X’Pert Pro MRD: PANalytical). For chemical analysis of the perovskite films, wavelength-dispersive x-ray fluorescence (WDXRF, XRF-1800: Shimadzu) using Rh *Kα* as x-ray source (20.216 keV) with analyzing single crystals of LiF (200) and Ge (111) was carried out. The morphologies of the perovskite films were observed using a field-emission scanning electron microscope (FE-SEM, Merlin-Compact: Carl Zeiss). The absorptance was measured by a UV-Vis spectrophotometer (V-770: JASCO). Steady-state and time-resolved photoluminescence spectra (LabRAM HV Evolution: Horiba, FluoTime 300: Picoquant) were obtained with excitation wavelength of 532 and 398 nm, respectively. The films were prepared on glass substrates with the incident light direction on the perovskite surface. The photocurrent-voltage (*J-V*) characteristics of the solar cells were obtained using a solar simulator (K3000: McScience, AM 1.5 G, 100 mW/cm^2^), with an active area of 0.09 cm^2^ and 100-mV/s voltage scan rate. The external quantum efficiency (EQE) spectra of the solar cells were measured by an incident photon-to-current conversion efficiency (IPCE) measurement system (K3100: McScience). Impedance analysis in dark condition was carried out using a potentiostat (Zive SP1: WonATech CO., Ltd.) to obtain frequency dependent capacitances with 10-mV amplitude of ac signal at zero applied bias and frequency ranging from 0.01 to 10^5^ Hz.

## Supplementary information

Supporting Information
