# Supplementary material for: Microstructural Evolution of Hybrid Perovskites Promoted by Chlorine and its Impact on the Performance of Solar Cell
Source: Sci Rep. 2019 Mar 18;9:4803. doi: 10.1038/s41598-019-41328-5 (PMC6423327; doi:10.1038/s41598-019-41328-5)
Supplement: Supplementary file 1 — Supporting Information [file 41598_2019_41328_MOESM1_ESM.pdf]

Supporting Information

Microstructural Evolution of Hybrid Perovskites Promoted by Chlorine and its Impact on the Performance of Solar Cell

Byungho Lee,[a] Taehyun Hwang,[a] Sangheon Lee,[a] Byungha Shin,[b,*] and Byungwoo Park[a,*]

[a] Department of Materials Science and Engineering, Research Institute of Advanced Materials, Seoul National University, Seoul 08826, Korea

[b] Department of Materials Science and Engineering, Korea Advanced Institute of Science and Technology, Daejeon 34141, Korea

Table S1. Photovoltaic parameters of the solar cells (more than 11 cells). Perovskites based on the various ratios of MACl to $MAPbI_3$ in precursors. The data in the parentheses are from the highest efficient devices. *HI*, η_{REV}, and η_{FOR}, are the hysteresis index, average photovoltaic efficiencies at reverse and forward scans, respectively.

Sample	J_{sc} (mA/cm²)	V_{oc} (V)	*FF*	η (%)	*HI* ($1-\eta_{FOR}/\eta_{REV}$)
**0 mol. % MACl (*REV*)**	20.3 ± 1.1 (21.4)	0.98 ± 0.03 (1.03)	0.69 ± 0.05 (0.76)	13.8 ± 2.1 (16.7)	0.21
**0 mol. % MACl (*FOR*)**	20.4 ± 1.1 (21.3)	0.88 ± 0.06 (0.92)	0.60 ± 0.09 (0.66)	10.9 ± 2.6 (13.0)	
**20 mol. % MACl (*REV*)**	21.3 ± 0.5 (21.3)	1.01 ± 0.02 (1.02)	0.77 ± 0.04 (0.81)	16.6 ± 1.0 (17.7)	0.05
**20 mol. % MACl (*FOR*)**	21.3 ± 0.5 (20.9)	0.97 ± 0.04 (1.01)	0.76 ± 0.04 (0.81)	15.7 ± 1.4 (17.1)	
**40 mol. % MACl (*REV*)**	21.4 ± 0.6 (21.5)	0.96 ± 0.02 (1.01)	0.72 ± 0.04 (0.79)	14.9 ± 1.1 (17.1)	0.03
**40 mol. % MACl (*FOR*)**	21.6 ± 0.5 (21.8)	0.92 ± 0.03 (0.99)	0.72 ± 0.04 (0.79)	14.4 ± 1.2 (17.0)	

* E-mail: byungha@kaist.ac.kr. Phone: +82-42-350-3315. Fax: +82-42-350-3310.
* E-mail: byungwoo@snu.ac.kr. Phone: +82-2-880-8319. Fax: +82-2-885-9671.

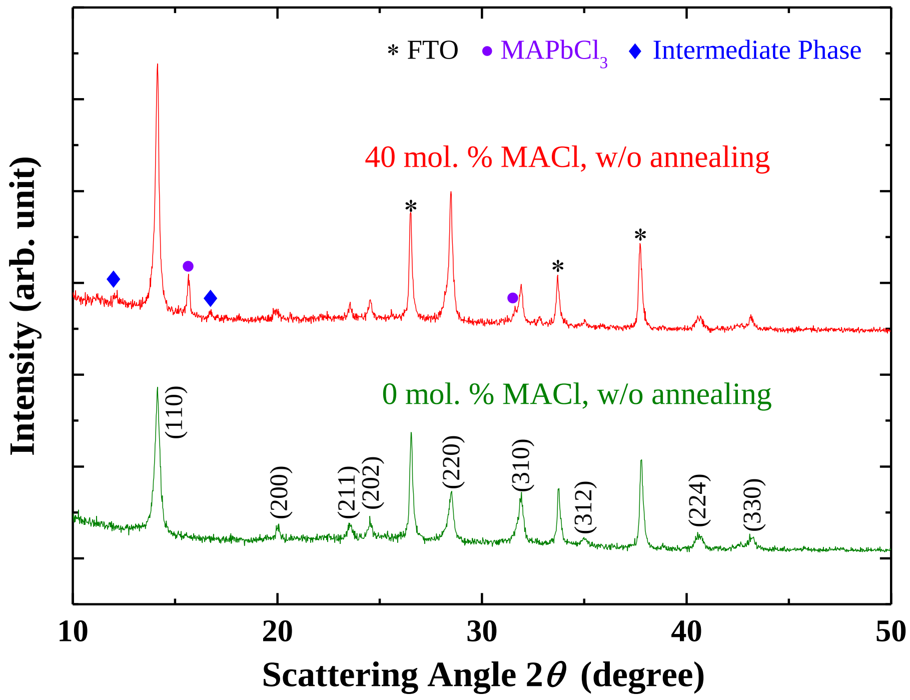

Fig. S1. X-ray diffraction of perovskite films without annealing.

Perovskites based on the MACl-containing precursor (40 mol. % MACl) and stoichiometric precursor (0 mol. % MACl).

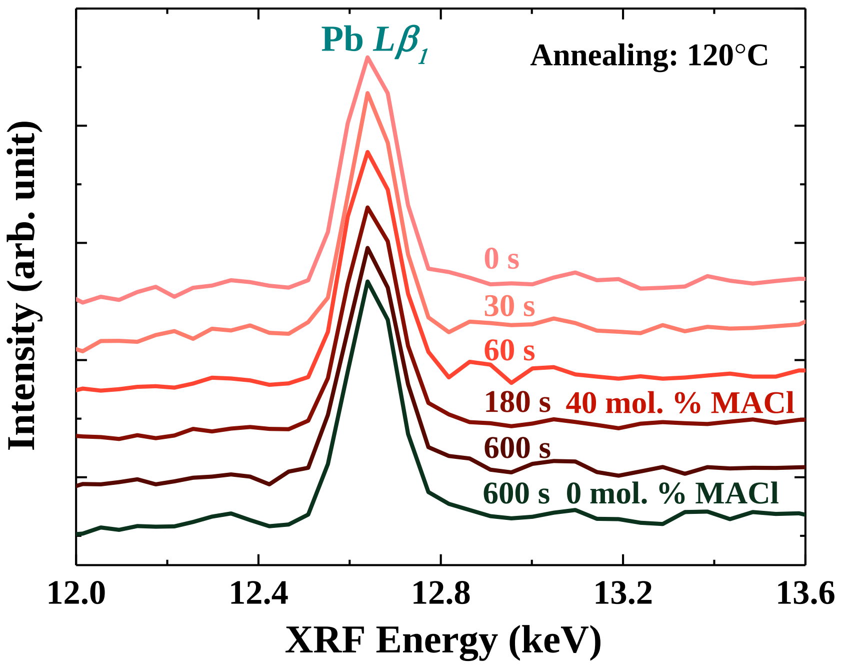

Fig. S2. **XRF spectra of Pb with different annealing times at 120°C.**
Perovskite films with MACl-containing precursor (40 mol. % MACl) and stoichiometric precursor (0 mol. % MACl).

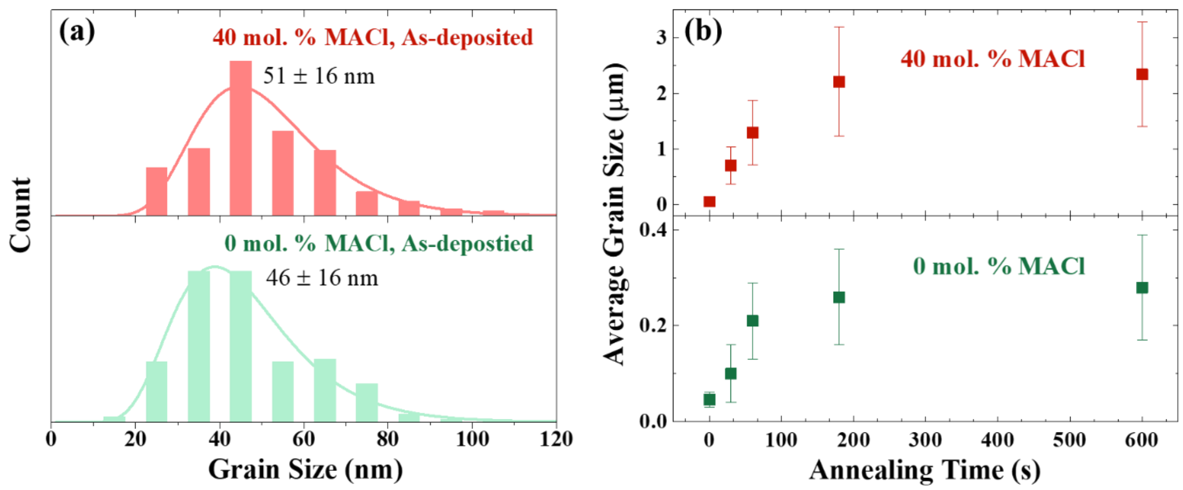

Fig. S3. Grain sizes of perovskite films with different annealing times.

(a) Distributions of grain sizes without annealing and (b) average grain sizes of the perovskites based on the MACl-containing precursor (40 mol. % MACl) and stoichiometric precursor (0 mol. % MACl).

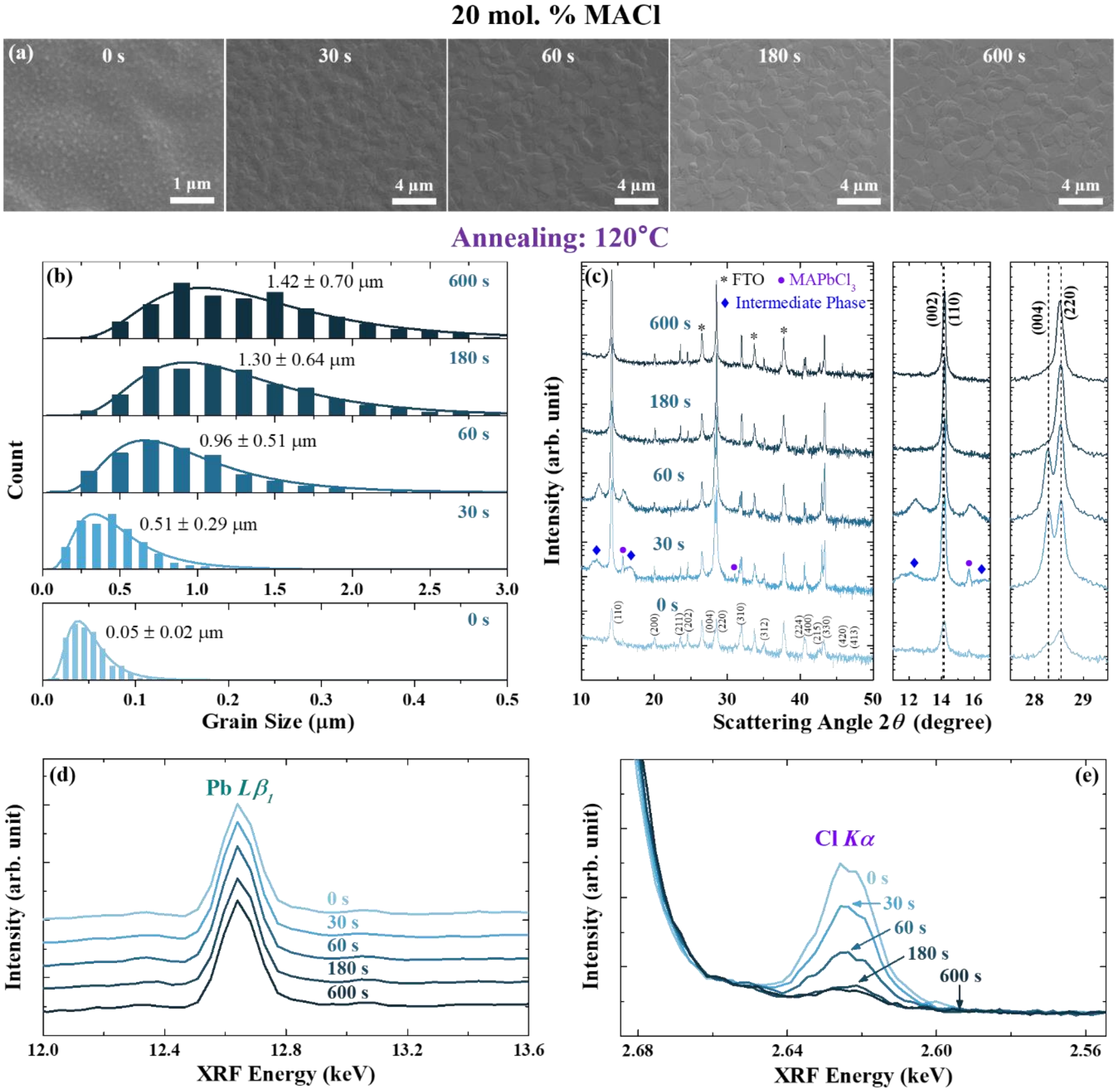

Fig. S4. Evolution of structural and chemical properties of perovskite films (20-mol.-% MACl) with annealing time.

(a) Grain growth (by SEM), (b) grain-size distribution with fitting lines, (c) x-ray diffraction, and (d,e) XRF spectra of Pb and Cl, respectively, for the perovskite films with different annealing time at 120°C.

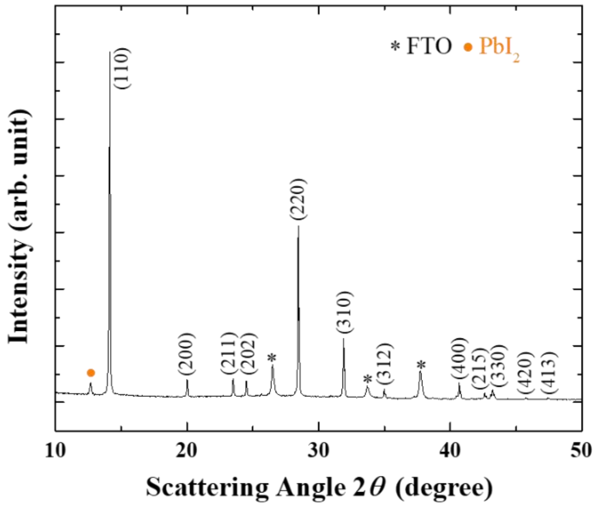

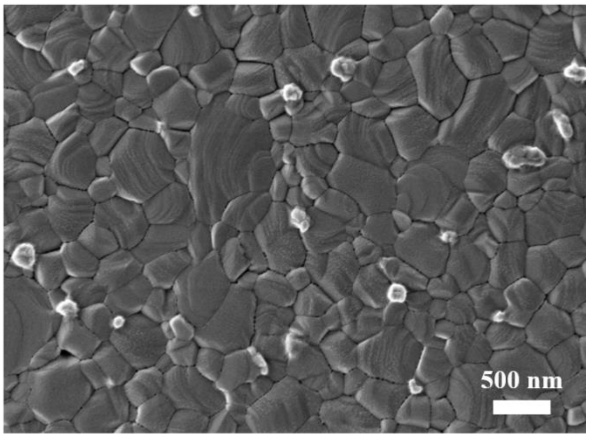

Fig. S5. Formation of PbI_2 in the perovskite films.

X-ray diffraction and SEM image of a perovskite with a stoichiometric precursor annealed at 120°C for 30 min. PbI_2 particles (bright region due to insulating nature) are formed at the grain boundaries.

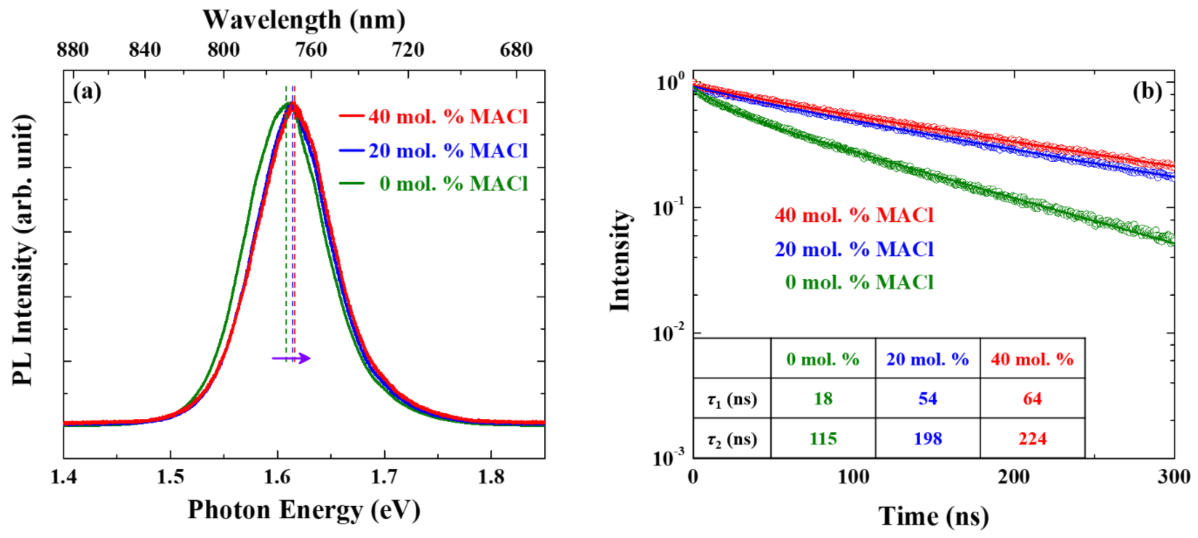

Fig. S6. Photoluminescence (PL) properties of the perovskite films.

(a) Steady-state PL spectra. Purple arrow indicates blue shift of emission peaks with increasing MACl contents in the precursors. (b) Time-resolved PL spectra with bi-exponential decay fitting (solid lines). Fast (τ_1) and slow (τ_2) components are listed in the inset.

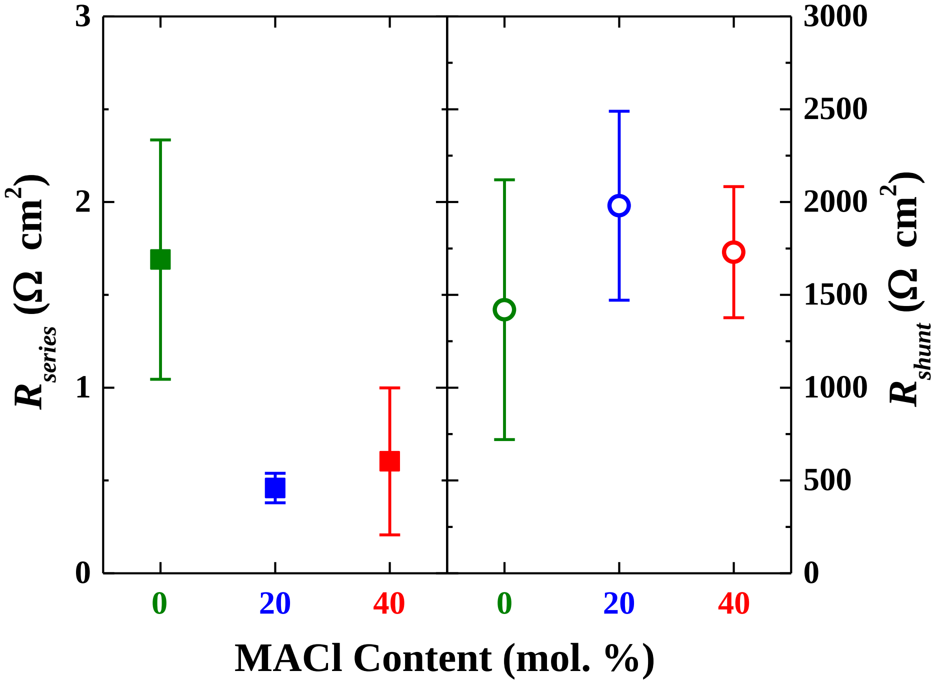

Fig. S7. **Series resistance (*R_{series}*) and shunt resistance (*R_{shunt}*) of the solar cells.**

The results are obtained by fitting the *J-V* curves with an ideal one-diode model.

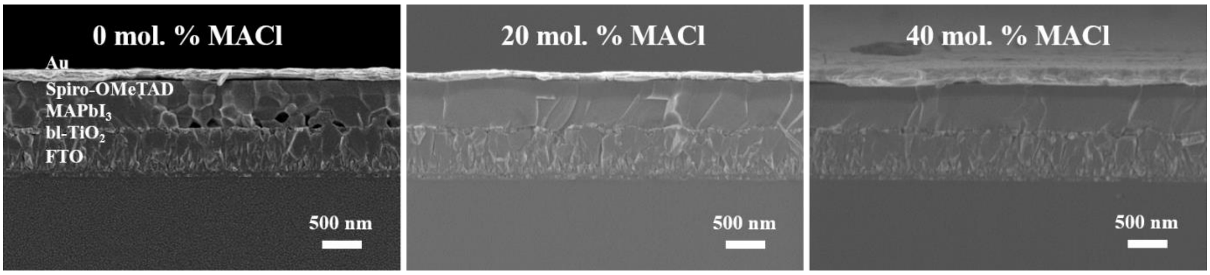

Fig. S8. **Cross-section images of perovskite cells (SEM).**

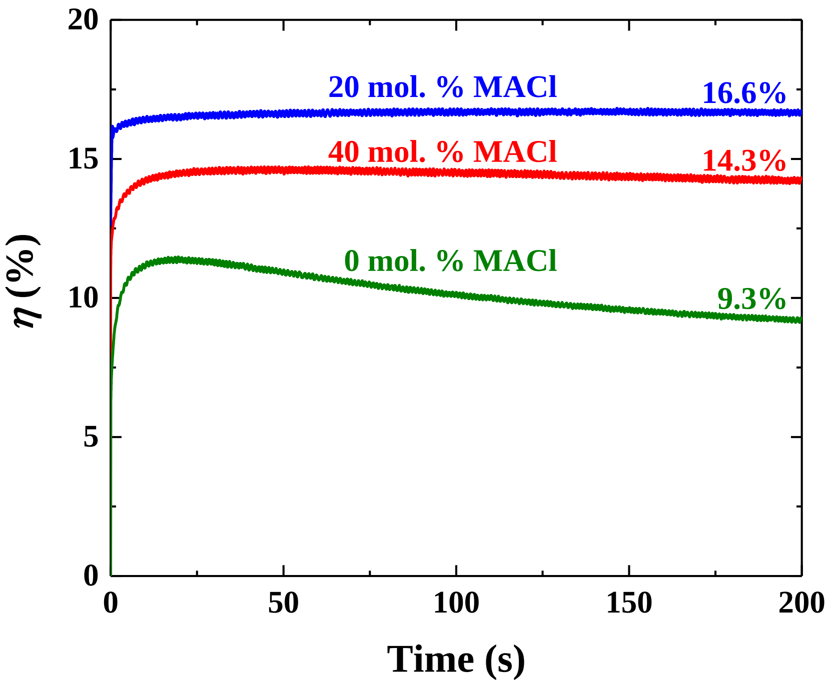

Fig. S9. **Stabilized power outputs of the perovskite solar cells.**

The power conversion efficiencies were measured with applied bias voltage at the maximum power point for each devices (0-mol.-% MACl: 0.70 V, 20-mol.-% MACl: 0.82 V, and 40-mol.-% MACl: 0.80 V).

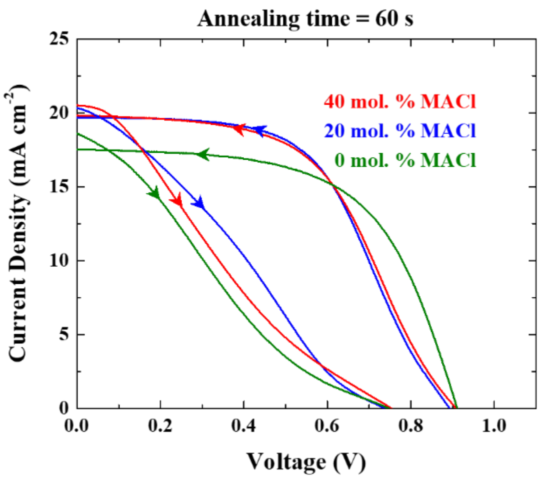

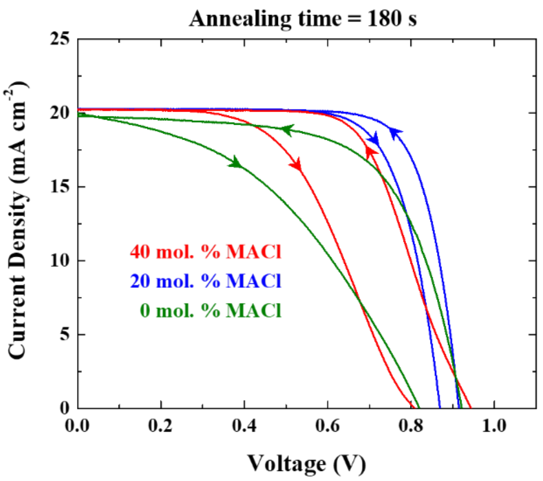

Sample	J_{sc} (mA/cm^2)	V_{oc} (V)	*FF*	η (%)
0 mol. % MACl (*REV*)	17.5 ± 0.6	0.91 ± 0.01	0.60 ± 0.01	9.6 ± 0.4
0 mol. % MACl (*FOR*)	18.5 ± 0.1	0.75 ± 0.01	0.23 ± 0.02	3.1 ± 0.2
20 mol. % MACl (*REV*)	19.7 ± 0.4	0.89 ± 0.03	0.55 ± 0.03	9.7 ± 0.6
20 mol. % MACl (*FOR*)	20.4 ± 0.5	0.74 ± 0.01	0.29 ± 0.04	4.3 ± 0.8
40 mol. % MACl (*REV*)	19.9 ± 0.5	0.90 ± 0.02	0.54 ± 0.08	9.7 ± 1.7
40 mol. % MACl (*FOR*)	20.5 ± 0.4	0.75 ± 0.02	0.23 ± 0.03	3.6 ± 0.6

Sample	J_{sc} (mA/cm^2)	V_{oc} (V)	*FF*	η (%)
0 mol. % MACl (*REV*)	19.9 ± 0.5	0.92 ± 0.03	0.65 ± 0.01	11.9 ± 0.8
0 mol. % MACl (*FOR*)	20.0 ± 0.3	0.82 ± 0.02	0.44 ± 0.03	7.1 ± 0.8
20 mol. % MACl (*REV*)	20.1 ± 0.4	0.92 ± 0.01	0.78 ± 0.01	14.4 ± 0.5
20 mol. % MACl (*FOR*)	20.2 ± 0.5	0.87 ± 0.01	0.75 ± 0.01	13.2 ± 0.4
40 mol. % MACl (*REV*)	20.2 ± 0.3	0.95 ± 0.01	0.66 ± 0.01	12.7 ± 0.2
40 mol. % MACl (*FOR*)	20.3 ± 0.5	0.81 ± 0.01	0.54 ± 0.01	8.8 ± 0.3

Fig. S10. **Solar cell performance with different annealing times at 120°C.**
J−V curves at both reverse and forward voltage scans with tables of the average photovoltaic parameters from more than 3 cells.

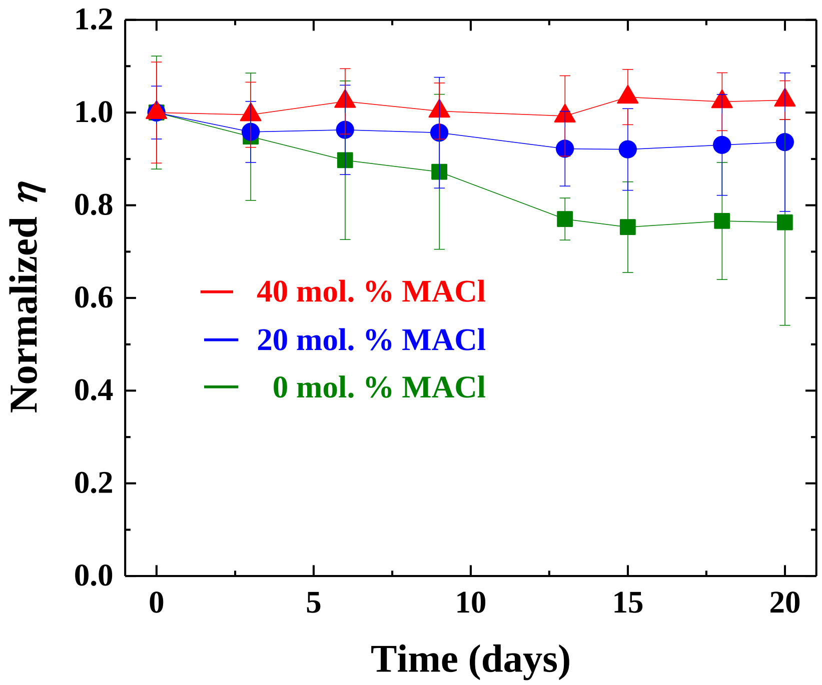

Fig. S11. **Stability test of the perovskite solar cells in air.**

The devices without encapsulation were stored in ambient air under dark condition (25°C and relative humidity < 20%). The power conversion efficiencies were extracted from the *J*-*V* curves with reverse scans.

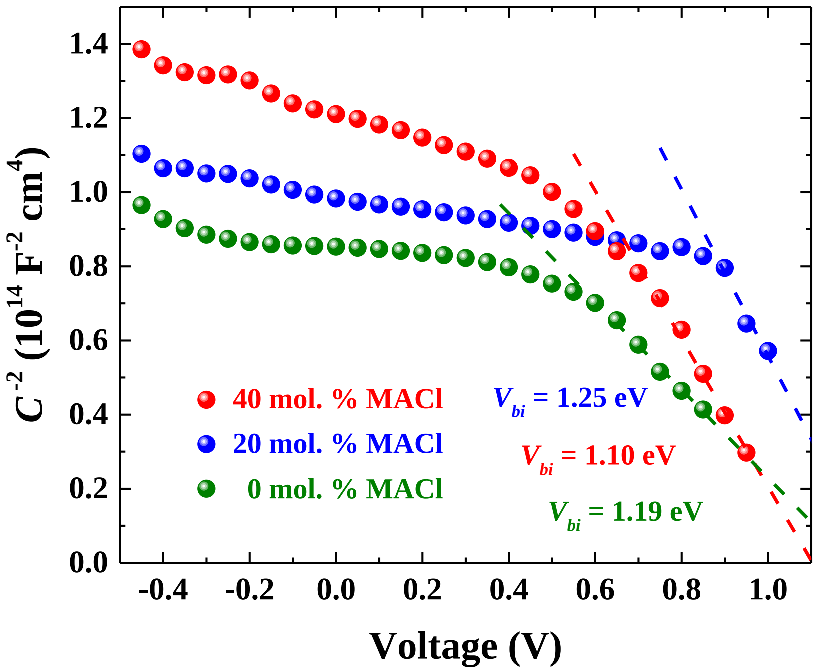

Fig. S12. Mott-Schottky plots of perovskite solar cells with various MACl concentration. The built-in potentials were extracted from the dashed fitting lines. The measurements were carried out under dark condition with 10-mV amplitude at 5 kHz.
